# Dichloroacetate and thiamine improve survival and mitochondrial stress in a *C*. *elegans* model of dihydrolipoamide dehydrogenase deficiency

**DOI:** 10.1172/jci.insight.156222

**Published:** 2022-10-24

**Authors:** Chynna N. Broxton, Prabhjot Kaur, Manuela Lavorato, Smruthi Ganesh, Rui Xiao, Neal D. Mathew, Eiko Nakamaru-Ogiso, Vernon E. Anderson, Marni J. Falk

**Affiliations:** 1 Mitochondrial Medicine Frontier Program, Division of Human Genetics, Department of Pediatrics, Children’s Hospital of Philadelphia, Philadelphia, Pennsylvania, USA.; 2 Department of Statistics and; 3 Department of Pediatrics, University of Pennsylvania Perelman School of Medicine, Philadelphia, Pennsylvania, USA.

**Keywords:** Genetics, Metabolism, Drug therapy, Mitochondria

## Abstract

Dihydrolipoamide dehydrogenase (DLD) deficiency is a recessive mitochondrial disorder caused by depletion of DLD from α-ketoacid dehydrogenase complexes. *Caenorhabditis elegans* animal models of DLD deficiency generated by graded feeding of *dld-1(RNAi)* revealed that full or partial reduction of DLD-1 expression recapitulated increased pyruvate levels typical of pyruvate dehydrogenase complex deficiency and significantly altered animal survival and health, with reductions in brood size, adult length, and neuromuscular function. DLD-1 deficiency dramatically increased mitochondrial unfolded protein stress response induction and adaptive mitochondrial proliferation. While ATP levels were reduced, respiratory chain enzyme activities and in vivo mitochondrial membrane potential were not significantly altered. DLD-1 depletion directly correlated with the induction of mitochondrial stress and impairment of worm growth and neuromuscular function. The safety and efficacy of dichloroacetate, thiamine, riboflavin, 5-aminoimidazole-4-carboxamide-1-β-d-ribofuranoside (AICAR), l-carnitine, and lipoic acid supplemental therapies empirically used for human *DLD* disease were objectively evaluated by life span and mitochondrial stress response studies. Only dichloroacetate and thiamine showed individual and synergistic therapeutic benefits. Collectively, these *C. elegans dld-1(RNAi)* animal model studies demonstrate the translational relevance of preclinical modeling of disease mechanisms and therapeutic candidates. Results suggest that clinical trials are warranted to evaluate the safety and efficacy of dichloroacetate and thiamine in human *DLD* disease.

## Graphical abstract

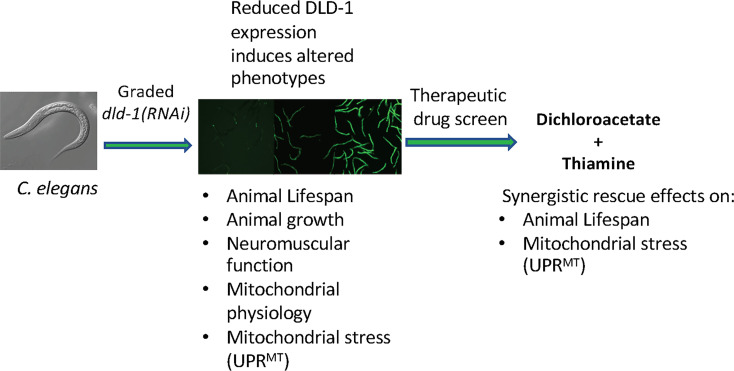

## Introduction

**Figure 1:**
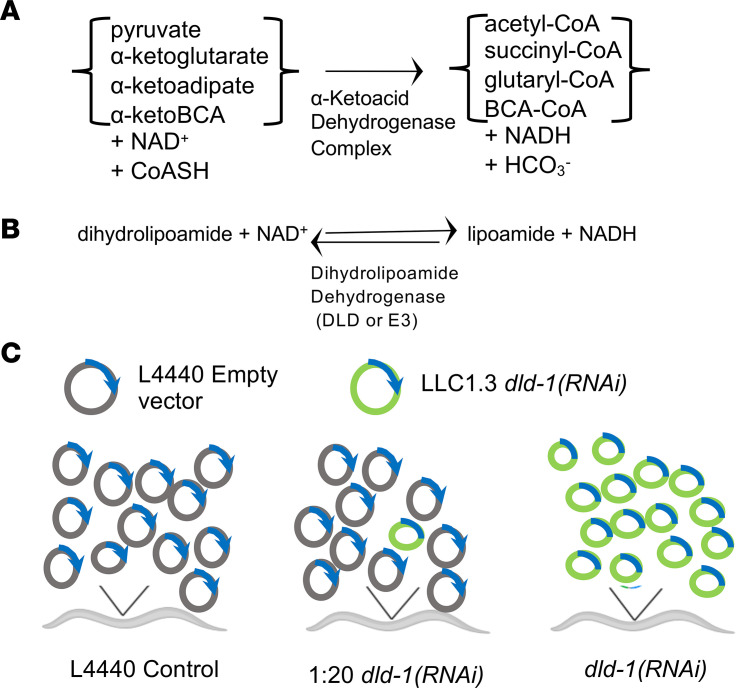
α-Ketoacid dehydrogenase catalyzed reactions and graded *dld-1(RNAi)* approach. (**A**) Overall oxidative decarboxylation reaction catalyzed by α‑ketoacid dehydrogenase complexes. The DLD-dependent α‑ketoacid dehydrogenase enzymes generate acyl-CoAs by oxidative decarboxylation of the identified α‑ketoacids coupled to the acylation of CoA with the concomitant production of bicarbonate (HCO_3_^–^) and the reduction of oxidized NAD^+^ to NADH. (**B**) DLD-1 (also referred to as E3) catalyzes the reversible oxidation of dihydrolipoamide by NAD^+^, a partial reaction of the overall reaction shown in **A**. (**C**) *dld‑1(RNAi)* model of graded DLD deficiency in *C*. *elegans*. Worms were grown on live *E*. *coli* harboring a plasmid under 2 T7 polymerases whose replication was induced by IPTG. The L4440 control N2 Bristol worms contained an empty vector, while the *dld-1*(*RNAi*) was present in plasmid LLC1.3. A reduced dose at 5% of full RNAi induction levels (middle) was achieved by feeding worms with diluted plasmid LLC1.3 at 1:20 ratio with the control L4440 vector.

Dihydrolipoamide dehydrogenase (DLD) deficiency is a rare, autosomal recessive, genetic disorder caused by pathogenic variants in *DLD*. *DLD* encodes a 54 kDa DLD protein in humans that serves as the E3 subunit of 4 mitochondrial matrix enzyme complexes (Figure 1A): pyruvate dehydrogenase complex (PDHc), α‑ketoglutarate dehydrogenase complex (KGDHc), α‑ketoadipate dehydrogenase complex (KADHc), and branched chain α-ketoacid dehydrogenase complex (BCKDHc) (1). DLD catalyzes the oxidation of dihydrolipoic acid bound to a lysine ε-amino group of the E2 component in all 4 of these enzyme complexes (Figure 1B) (2). PDHc couples glycolysis to the TCA cycle, KGDHc functions within the TCA cycle, KADHc is a mitochondrial enzyme in the lysine catabolism pathway, and all 4 reactions yield NADH that transfers the reducing equivalents to the mitochondrial respiratory chain (RC) to drive ATP formation. DLD has also been reported to function as the third component in the glycine cleavage system, in which it is known as protein L (3, 4).

*DLD* disease symptoms are multisystemic and may variably include elevated lactic acid levels, liver dysfunction, Leigh syndrome, Reye syndrome, developmental disabilities, neurological deficits including seizures and ataxia, cardiomyopathy, and failure to thrive. These disease symptoms can manifest early during the neonatal period or later during adulthood. To date, most *DLD* disease symptoms have been attributed to dysfunction of PDHc. In addition, deficiency of KGDHc has been identified as a significant contributor to mitochondrial reactive oxygen species (ROS) and implicated in *DLD* disease (5), and the heterozygous R263H mutation has been identified as a genetic risk factor for Alzheimer disease (6). However, full understanding of the relative mechanistic contributions of the different E3-containing mitochondrial enzyme complexes to human *DLD* disease phenotypes is still being elucidated. Deficiency in E3 activity would be anticipated to increase the concentration of the substrate α-ketoglutarate acids along with NAD^+^ and CoA while potentially reducing both NADH and acylated CoA species. Elevated levels of α-ketoglutarate in urine and branched chain amino acids in plasma suggest that dysfunction of KGDHc and BCKDHc may also contribute to *DLD* disease pathology (7). The detection of KGDHc in the nucleus has been shown to provide succinyl-CoA for the succinylation of histones (8), providing a potential connection between DLD deficiency and epigenetic dysregulation. An increase in α-ketoglutarate may also enhance the action of histone demethylases (9, 10).

High-resolution crystal structures of wild-type and pathogenic variants of human DLD offer molecular insight into the involvement of ROS in *DLD* disease pathology (5, 11–15). Deleterious ROS can be produced by the mutant E3 when it is dissociated from E1 and E2 in the intact PDH and KGDH complexes (16). ROS produced by these mechanisms are thought to exacerbate symptoms caused by dysfunction of E3-containing complexes. An alternative mechanism could be that the absence of E3 subunits destabilizes the complex structures, resulting in activation of the mitochondrial unfolded protein stress response (UPR^mt^) (17).

Introduction of homozygous null mutations into mouse *Dld* results in embryonic lethality, demonstrating the requirement for *Dld* during embryonic development (18). In contrast, mice harboring a heterozygous null *Dld* allele are phenotypically normal despite displaying half of the E3 amount and half of the E3-containing complex activities as observed in wild-type mice (18). While studies using *DLD* human disease patient fibroblast lines have demonstrated deficiencies in the activities of E3-containing complexes (19), no animal model that recapitulates the complex neurologic, developmental, cardiac, and hepatic phenotypes observed in patients with DLD deficiency has been reported to date. To establish translational models in which to better understand disease mechanism(s) and develop effective therapies, we utilized a *Caenorhabditis elegans* invertebrate animal feeding RNAi knockdown model of DLD deficiency.

Here, we report mechanistic investigations into the role of severe or partial DLD deficiency on animal survival and health span, as well as mitochondrial physiology, using a graded feeding RNAi approach in *C*. *elegans* (Figure 1C). Whole-animal effects were quantified at the levels of survival, development, growth, fecundity, and neuromuscular function. In addition, effects of DLD deficiency on diverse aspects of mitochondrial physiology were evaluated, including enzymatic activities of E3 and RC enzyme complexes; levels of ATP, pyruvate, and lactate; and in vivo mitochondrial content, membrane potential, and mitochondrial stress induction. Subsequently, drug screens were performed on animal survival and mitochondrial stress induction to objectively quantify effects of standard-of-care or predicted mitochondrial therapies used on an empiric basis in human patients with *DLD* disease (7). These results suggested that combination therapy of dichloroacetate (DCA) and thiamine provided the most significant improvement in organismal health in *DLD* disease.

## Results

### Confirmation of DLD protein and activity deficiency in C. elegans RNAi models.

The nematode *C*. *elegans* harbors a single *dld* gene encoding DLD-1, a protein that is 72% identical in amino acid sequence to human DLD. Sequence alignment of DLD from humans with DLD-1 from *C*. *elegans* shows high conservation of the mitochondrial targeting sequence, flavin adenine dinucleotide (FAD) binding domain, NAD binding domain, as well as central and interface domains, which are all necessary for DLD function (Supplemental Figure 1; supplemental material available online with this article; https://doi.org/10.1172/jci.insight.156222DS1). Thus, it is likely that DLD performs similar functions in both organisms, supporting the use of *C*. *elegans* to model relevant translational phenotypes of DLD deficiency.

**Figure 2:**
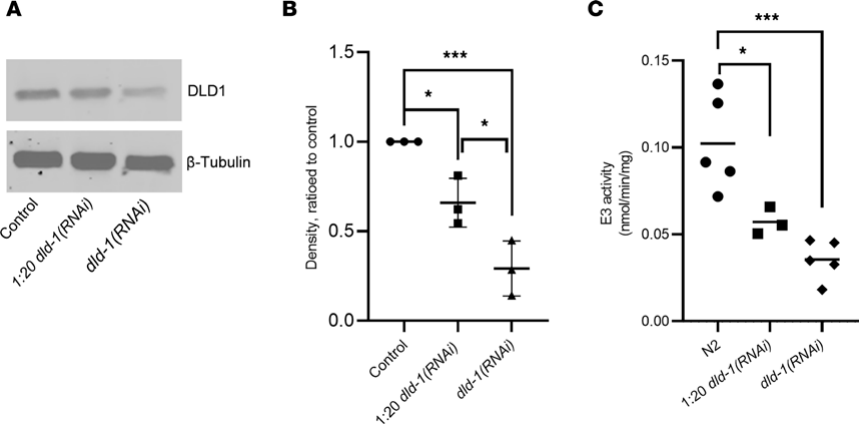
*dld-1(RNAi)* worms displayed reduced DLD-1 protein expression and E3 enzyme activity. (**A**) Representative DLD-1 Western blot. DLD-1 expression was assayed by immunoblotting after exposure to *dld-1(RNAi)* at partial or full dose. (**B**) DLD-1 protein expression quantitation. Fluorescence scans of the blots were quantified in ImageJ (NIH). RNAi expression level was normalized first to β‑tubulin as a loading control and then to wild-type (N2) control expression level. DLD-1 expression was reduced by 38% and 71% in the 1:20 dilution and undiluted *dld-1(RNAi)*, respectively. Error bars reflect the SD of 3 biological replicates. The results were analyzed by 2-sided 1-way ANOVA, followed by Tukey’s multiple comparisons; *P < 0.05, ***P < 0.001. (**C**) DLD-1 (E3) enzyme activity. One thousand worms were homogenized per replicate for biochemical assays of dihydrolipoamide dehydrogenase activity. For biological replicates, N2 and RNAi‑treated worms were grown in parallel. E3 activity was decreased by 45% and 66% for the 1:20 dilution and undiluted *dld‑1(RNAi)*, respectively. The results were analyzed by 2-sided 1-way ANOVA, followed by Tukey’s multiple comparisons, *P < 0.05, ***P < 0.001.

Mutations resulting in deletion of *dld-1* are embryonic lethal in *C*. *elegans*, as annotated by the Japanese worm consortium (20). Therefore, we utilized feeding RNAi to variably deplete DLD-1 protein in N2 Bristol wild-type worms (N2). Depletion of DLD-1 was achieved by bacterial feeding of worms with the RNAi clone LLC1.3 (https://wormbase.org/search/gene/LLC1.3) (21), which encodes a dsRNA against *dld-1* when induced by isopropyl-β-d-thiogalactoside (IPTG) that reduces DLD-1 expression in *C*. *elegans* (22). Previous work demonstrated that graded feeding of this *dld-1(RNAi)* led to variation in animal phenotype depending on the amount of RNAi expressed (23). Therefore, we followed a similar approach to model human DLD deficiency in *C*. *elegans*. To confirm DLD-1 knockdown, worms were grown from the stage of eggs laid on bacteria harboring control RNAi (L4440), a 1:20 dilution of *dld-1(RNAi)* with control RNAi, or *dld-1(RNAi)* in the presence of 0.2 mM IPTG. Synchronized populations of young-adult-stage worms were grown, and lysates were prepared. Western immunoblots (Figure 2A, full gel in online supplement) revealed 38% (*P* < 0.05) and 71% (*P* < 0.001) DLD-1 protein knockdown was achieved, respectively, in the worms fed the 1:20 *dld‑1(RNAi)* dilution or undiluted (full-dose) *dld-1(RNAi)* (Figure 2B). Enzymatic analysis of DLD-1 (E3) activity demonstrated in 1:20 RNAi dilution a 45% decrease (*P* < 0.05) and in full-dose RNAi a 66% (*P* < 0.001) decrease in E3 activity in DLD-1–depleted animals as compared with N2 (Figure 2C), similar to the degree of E3 deficiency observed in human disease studies (14). Therefore, RNAi knockdown of DLD-1 leads to a viable *C*. *elegans* model with significant DLD-1 protein deficiency with substantially reduced E3 activity in adult worms.

### DLD-1 deficiency reduces animal growth and brood size in C. elegans.

**Figure 3:**
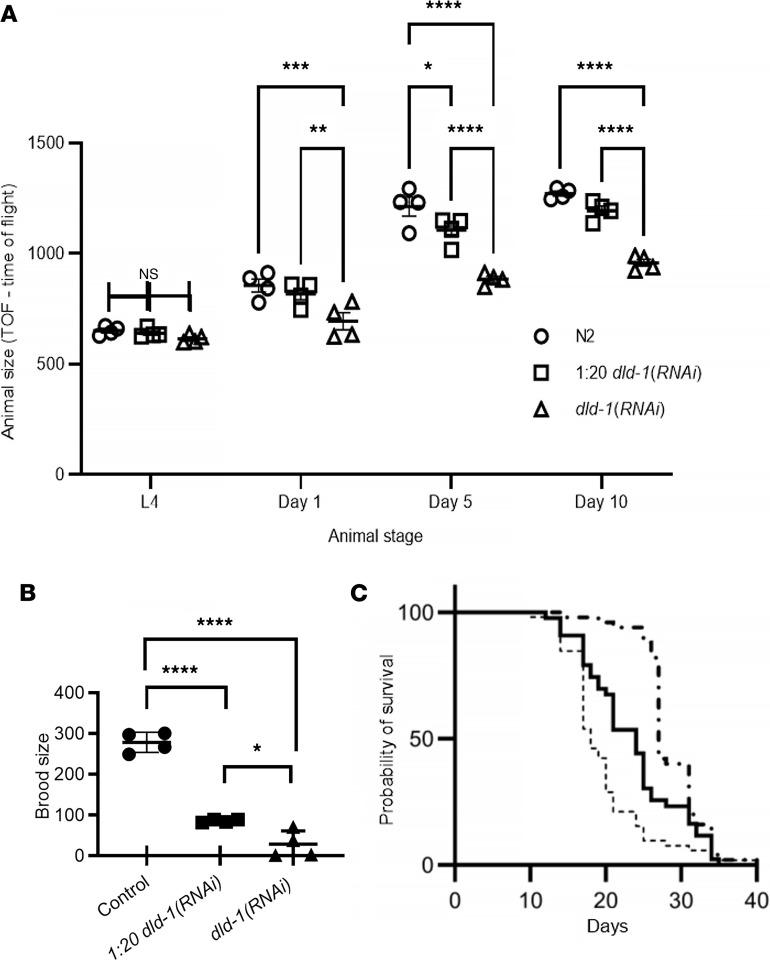
DLD-1–deficient worms had reduced growth and brood size, with altered survival. (**A**) Adult growth of *dld-1*(*RNAi*) worms was diminished. While L4 worms displayed no difference in size, Circles: control worms, squares: 1:20 diluted *dld-1(RNAi)*, triangles: full-dose *dld-1(RNAi)*. Adult DLD-1–deficient worms, both the diluted and full dose, had reduced linear growth. At adult day 1, the full-dose worms were significantly shorter than either the 1:20 diluted-dose or control worms (**P < 0.01 and ***P < 0.001, respectively) while at adult day 5, the diluted-dose worms were 10% shorter and the full-dose worms 25% shorter than control (*P < 0.05 and ****P < 0.0001, respectively, determined by 2-way ANOVA followed by Tukey’s multiple comparisons). The reduced growth continued through adult day 10. Data points are the means ± SEM of 4 biological replicates. (**B**) Brood size of *dld-1*(*RNAi*) worms was reduced. Individual worms were separated and allowed to lay eggs, after which viable larvae were counted for 5 days. Brood size was severely decreased by more than 90% in DLD-1–knockdown worms relative to wild-type controls (N2). Bars convey mean and SD. The results were analyzed by 2-sided 1-way ANOVA, followed by Tukey’s multiple comparisons; *P < 0.05, **P < 0.01, ****P < 0.0001. (**C**) Differential effects on survival were observed with partial and full knockdown of *dld-1* by RNAi in *C*. *elegans*. Life span analysis was concurrently performed in L4440 control (solid line), 1:20 *dld-1*(*RNAi*) (dashed line, **- - -**), and *dld‑1*(*RNAi*) (alternating dash and dot line, **- · - · -**) worms at 20°C. Log-rank (Mantel-Cox) tests indicated partial DLD-1 knockdown decreased survival (*P* < 0.01, HR = 1.7) while full DLD-1 knockdown resulted in increased lifetime in 10 biological replicates (*P* < 0.0001, HR = 0.32). A single representative trial of 5 replicates is pictured.

Previous work has demonstrated that RNAi knockdown of DLD-1 results in a shorter final adult worm length than controls (23). Reduced worm length could be caused by delayed larval development and/or an ongoing growth defect that occurs during early adulthood. Indeed, we observed that worms grown on either *dld-1(RNAi)* or control RNAi from hatching proceeded through all L1–L4 larval developmental stages at the same rate, with no difference in animal length seen at the L4 larval stage (Supplemental Figure 2). As *C*. *elegans* normally undergo a 70% increase in worm length between day 1 and day 14 of adulthood (24), we sought to determine whether defects in adult growth underlie the length deficits observed with partial or full-dose *dld-1(RNAi)* feeding. To obtain the lengths of worms from a synchronous population whose growth was followed from L4 to adult day 10, on adult days 1, 5, and 10, a random selection of approximately 100–300 worms from the control and the reduced- and full-dose *dld-1(RNAi)* cohorts in 4 biological replicates were analyzed by flow cytometry (BioSorter, Union Biometrica). A typical sampling of the worms was arranged in groups and photographed (Supplemental Figure 2). In contrast to worms grown on control RNAi in which length increased between L4 stage and adult day 5 by 86% and between L4 stage and adult day 10 by 95%, DLD-1–deficient worms induced by full-dose RNAi at day 5 and day 10 displayed only a 44% and 56% mean increase in body length, respectively (Figure 3A). This translated to adult day 5 and day 10 DLD-1–deficient worms being approximately 27% shorter than N2 worms, without substantial linear growth in DLD-1–deficient animals observed between adult days 5 and 10 (Figure 3A, *P* < 0.0001). This effect was dose dependent, as the worms fed 1:20 diluted *dld-1*(*RNAi*) were intermediate in growth at adult days 1, 5, and 10.

Perturbations in mitochondrial proteins either through genetic mutation (25) or RNAi often cause decreased fecundity with reduced brood size in *C*. *elegans* (26). Therefore, we investigated whether feeding RNAi depletion of DLD-1 for 1 generation impacted worm reproduction. DLD-1–depleted and N2 worms were grown from the L1 to L4 stage, after which progeny from each adult hermaphrodite worm were counted over 5 days. A dramatic decrease of 90% in mean brood size was observed in full-dose DLD-1–depleted worms in 4 biological replicates (Figure 3B, *P* < 0.0001). Further, even with the 1:20 diluted *dld1(RNAi)*, a greater than 50% decrease in brood size was observed (*P* < 0.0001). Therefore, partial- or full-dose DLD-1 deficiency leads to significant reduction of both adult linear growth and animal fecundity in *C*. *elegans*.

### DLD-1 deficiency modulates life span in C. elegans.

A common phenotype in genetic mutations that impact mitochondrial homeostasis is a change in overall adult survival (27). As mitochondrial mutant *C*. *elegans* have been identified that can lead to either a shortened or extended life span, we investigated the impact of DLD-1 deficiency on animal life span. Survival was monitored manually in synchronous worm populations grown from egg hatching on control RNAi, 1:20 *dld-1(RNAi)* dilution, or full-dose *dld-1(RNAi)*. Life span analysis of worm populations grown on *dld-1(RNAi)* were consistent with the previous report (23). Specifically, a significantly *decreased* median life span by –4.8 ± 0.8 days was observed in 10 separate biological replicate experiments when worms were grown from L1 stage on a 1:20 dilution of *dld-1(RNAi)* compared with worms grown on L4440 control bacteria (Figure 3C). However, a significantly *increased* median life span of by 4.5 ± 0.6 days was observed in these same 10 replicate experiments when worms were grown on the full dose of *dld-1(RNAi)* from L1 stage as compared with N2 worms grown on L4440 *E*. *coli* (*P* < 0.0001) (Figure 3C). These data support that a moderate reduction of DLD-1 expression levels achieved through feeding a 1:20 dilution of *dld-1(RNAi)* decreases animal length, fecundity, and life span, whereas a more severe reduction of DLD-1 protein achieved with full-dose *dld-1(RNAi)* causes greater adult growth and fecundity inhibition but notably increased animal survival.

### DLD-1 deficiency impairs neuromuscular function in C. elegans.

**Figure 4:**
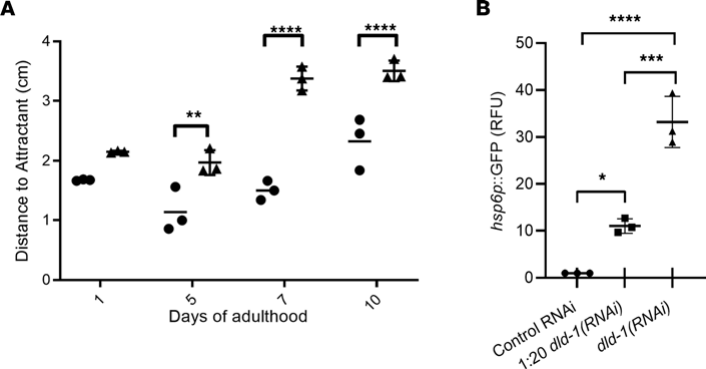
DLD-1–deficient worms displayed impaired neuromuscular behavior and increased mitochondrial stress. (**A**) Chemotaxis assay. Worms fed *dld-1*(*RNAi*) developed impaired neuromuscular activity with age at the level of chemotaxis. Worms were placed 5 cm from a chemoattractant and allowed to move freely for 1 hour. While on adult day 1 most of both N2 (circles) and full-dose *dld-1(RNAi)* (triangles) worms were within 1.5 cm of the chemoattractant, on days 5, 7, and 10 the worms’ ability to move toward the chemoattractant had diminished to the point that the motion of the *dld-1(RNAi)* knockdown worms was nearly random. Points indicate the average animal distance (*n* = ~60 worms per condition) from the chemoattractant as measured in centimeters from 3 technical replicates. Error bars convey SD. Two-way ANOVA followed by Tukey’s multiple comparisons indicated the difference in mean distance was significant, with **P < 0.01 at day 5 and ****P < 0.0001 at both day 7 and day 10. (**B**) UPR^mt^ analysis. *dld-1(RNAi)* worms had increased mitochondrial stress with basal induction of the UPR^mt^. Worms subjected to both 1:20 diluted and full-strength *dld‑1(RNAi)* displayed significant induction of *hsp-6p*:GFP fluorescence, indicating the presence of the UPR^mt^ response occurred under basal growth conditions. Each condition was assessed in 3 biological replicates with *n* = 200 worms per trial. Error bars convey SEM. The results were analyzed by 2-sided 1-way ANOVA, followed by Tukey’s multiple comparisons; *P < 0.05, ***P < 0.001, ****P < 0.0001.

Human DLD deficiency is characterized by a range of neurological defects and impaired coordination (7). To determine whether depletion of DLD-1 impaired integrated neuromuscular function in *C*. *elegans*, we utilized a validated chemotaxis assay to test the ability of worms to sense and crawl over 1 hour toward isoamyl alcohol, a diffusive chemoattractant (28). On day 1 of adulthood, no significant difference was seen between control and full-dose *dld-1(RNAi)* fed worms in their ability to crawl toward the chemoattractant (Figure 4A). However, by adult day 4, DLD-1–deficient worms displayed a trend toward diminished ability to move toward the chemoattractant, which increased in severity as the worms aged such that on adult day 7 the DLD-1–deficient worms were on average 51% farther away from the chemoattractant than controls (*P* < 0.0001) (Supplemental Figure 3). Worms maintained on the 1:20 dilution of *dld-1(RNAi)* did not show significant impairment of chemotaxis (data not shown). Collectively, these data demonstrate that DLD-1–deficient worms displayed abnormal neuromuscular function, providing a quantitative behavior that serves as a proxy for the neuromuscular deficiencies commonly observed in human mitochondrial *DLD* disease.

### DLD-1–deficient worms exhibit altered mitochondrial physiology and increased UPR^mt^.

As neuromuscular defects associated with aging can be induced by mitochondrial stress, we investigated whether DLD-1–deficient worms showed objective evidence of mitochondrial stress. The UPR^mt^ is a pathway through which mitochondria undergoing proteotoxic stress signal to the nucleus to upregulate expression of nuclear genes that attempt to restore mitochondrial proteostasis, including protein chaperones (heat shock proteins) and antioxidants (29, 30). In particular, heat shock protein 6 (HSP6, ortholog of human HSPA9) is a protein chaperone induced by the UPR^mt^ response that has been previously used as a reporter gene in *C*. *elegans* to evaluate activation of this mitochondrial stress pathway (31). Here, we evaluated whether the UPR^mt^ was induced by RNAi knockdown of DLD-1 in the *hsp6p*:*gfp* *C*. *elegans* strain, which expresses inducible GFP under the *hsp6* promoter (*hsp6p)* only upon induction of mitochondrial stress (32). Specifically, we measured UPR^mt^ induction in *hsp6p*:GFP-expressing worms at day 2 of adulthood after having been grown from the L1 larval stage either on 1:20 dilution of *dld-1(RNAi)* or full-dose *dld-1(RNAi)*, with respective 11-fold and 33-fold UPR^mt^ induction as measured by GFP fluorescence when normalized to worms grown on control RNAi (Figure 4B, *P* < 0.001 and *P* < 0.0001, respectively). Collectively, these data demonstrate that mitochondrial stress induction correlates with E3 enzyme deficiency, linear growth failure, reduced progeny, and altered survival in DLD-1–deficient worms.

**Figure 5:**
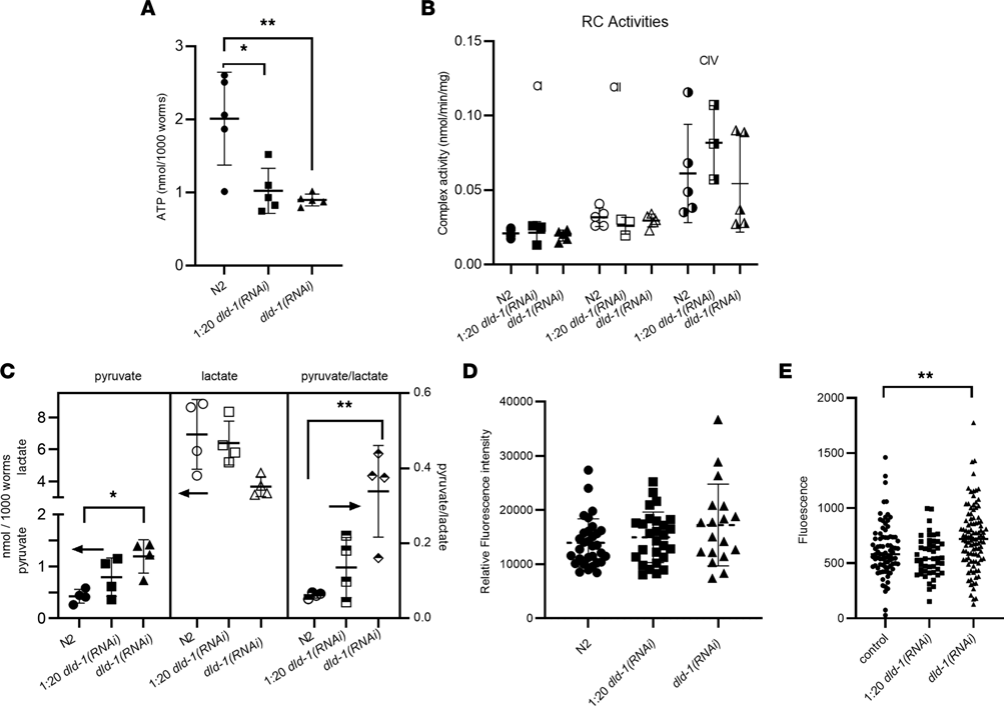
DLD-1–deficient worms displayed significantly reduced levels of ATP, increased levels of pyruvate and mitochondria, and normal RC function. For all mitochondrial experiments, worms were grown on diluted 1:20 *dld-1(RNAi)* (squares) and full-dose *dld-1(RNAi)* (triangles), where synchronized populations of approximately 1,000 worms were homogenized and assayed for different biochemical properties relative to wild-type (N2) worms (circles). Error bars display mean ± SD. (**A**) ATP. ATP amount was significantly decreased by *dld-1(RNAi)* in both the 1:20 partial and the full knockdown worms. The results were analyzed by 2-sided 1-way ANOVA, followed by Tukey’s multiple comparisons (*P < 0.05, **P < 0.01). (**B**) RC enzyme activities. RC enzyme activities of complexes I (CI, filled shapes), II (CII, unfilled shapes), and IV (CIV, half-filled shapes) were not significantly different in 1:20 or full-dose *dld-1(RNAi)* worms relative to wild-type (N2) control worms. (**C**) Pyruvate and lactate. Pyruvate (filled shapes) was significantly increased by the full-dose *dld-1(RNAi)* (*P < 0.05), while the lactate (unfilled shapes) level showed a decreasing trend resulting in a significant increase in the pyruvate/lactate ratio (half-filled shapes) (**P < 0.01). The results were analyzed by 2-sided 1-way ANOVA, followed by Tukey’s multiple comparisons. The arrows indicate the appropriate *y* axis for each data set. (**D**) Mitochondrial membrane potential. Integrated RC function at the level of in vivo mitochondrial membrane potential determined by the mitochondrial retention of TMRE normalized to COX4:GFP, as a proxy for mitochondrial content, was not significantly different in either 1:20 diluted or full dose *dld‑1(RNAi)* worms relative to wild-type (N2) controls. (**E**) Mitochondrial content. Significantly increased mitochondrial content was detected in the full *dld-1(RNAi)* knockdown worms by BioSorter analysis of *myo-3p*:GFP(mit) fluorescence. Scatterplot conveys means ± SEM, **P < 0.01 determined by 1-way ANOVA.

Given that DLD-1–deficient worms exhibited several phenotypes commonly seen in other models of mitochondrial dysfunction, we investigated whether their observed phenotypic abnormalities correlated with quantifiable defects in mitochondrial physiology. A vital function of the mitochondria is the aerobic production of cellular energy as ATP through oxidative phosphorylation. Therefore, we assessed total cellular ATP levels as well as enzymatic function of RC complexes I, II, and IV to determine whether DLD deficiency directly impaired mitochondrial energy production capacity. Synchronous populations of young adult worms grown on control or *dld-1(RNAi)* were harvested, and whole animal population lysates were prepared for biochemical analysis. Compared with worms grown on control RNAi, DLD-1 knockdown with a 1:20 dilution of *dld-1(RNAi)* or full-dose *dld-1(RNAi)* resulted in a 49% (*P* < 0.05) and 55% (*P* < 0.01) decrease, respectively, in total worm ATP levels (Figure 5A). Enzymatic activities of complexes I and II were unchanged in DLD-1–knockdown worms relative to control. A trend toward 11% reduced complex IV activity was seen in *dld‑1(RNAi)* worms compared with N2, which reached only marginal statistical significance (*P* = 0.06) (Figure 5B). We also quantified pyruvate and lactate levels in whole worm lysates, since pyruvate is classically elevated in individuals with PDHc deficiency (1). Indeed, pyruvate levels were increased by 2.8-fold in worms grown on full-dose *dld‑1(RNAi)* (*P* < 0.05) and by 1.9-fold in worms grown on 1:20 diluted *dld-1(RNAi)* relative to N2 control animals (Figure 5C). Conversely, tissue lactate level was decreased by 1.9-fold in the worms grown on *dld-1(RNAi)* and not significantly altered in 1:20 diluted *dld-1(RNAi)* relative to N2 controls (Figure 5C). As DLD-1 deficiency directly reduces activity of the α-ketoacid dehydrogenase, and hence the rate of NAD^+^ consumption, the resulting 3.8-fold increase observed in the pyruvate/lactate ratio of worms grown on full-dose *dld-1(RNAi)* relative to controls (*P* < 0.05) is consistent with the anticipated increase in levels of NAD^+^. Indeed, the resultant increase in the NAD^+^/NADH ratio and a more oxidative intracellular environment dependent on the degree of DLD-1 deficiency will occur if the lactate dehydrogenase reaction is near equilibrium.

DLD-1 deficiency’s impact on broader in vivo mitochondrial physiology in living worms was assessed by high-content imaging (CX5, Thermo Fisher Scientific) to quantify relative changes in mitochondrial membrane potential and BioSorter flow cytometry to quantify muscle mitochondrial content. All experiments utilized synchronized adult day 1 worms that were grown from the L1 larval stage on control RNAi, 1:20 dilution of *dld-1(RNAi)*, or full-dose *dld-1(RNAi)*. An in vivo measurement of the overall function of the mitochondrial RC is the determination of the electrochemical gradient generated by the process of oxidative phosphorylation across the inner mitochondrial membrane, where mitochondrial membrane potential serves as the transformer between oxidative metabolism and ATP production. To quantify mitochondrial membrane potential, COX4:GFP worms, *cox-4(zu476[cox-4:eGFP:3xFLAG])* (17), that carry a GFP marker in the COX4 complex IV subunit were used to simultaneously quantify mitochondrial content when animals were exposed to tetramethylrhodamine ethyl ester (TMRE) fluorescence dye used to quantify relative mitochondrial membrane potential. The relative membrane potential was determined from the ratio of TMRE fluorescence to GFP fluorescence. Indeed, DLD-1–deficient worms retained their ability to generate a membrane potential (Figure 5D and Supplemental Figure 4), consistent with the observed invariant activities of the RC complex enzymes as interrogated by spectrophotometric enzyme assays (Figure 5B). Thus, we postulate that rather than an inherent defect in integrated RC capacity, the observed decrease in tissue ATP levels in DLD-1–deficient worms results at least in part from decreased NADH production that reduces the availability of electrons to feed into the RC.

Mitochondrial dysfunction that involves decreased ATP production can lead to adaptive alterations in mitochondrial content (33, 34). Indeed, in 1 case where muscle biopsy tissue was examined, a patient with *DLD* disease was shown to have mitochondria proliferation in muscle tissue (35). Therefore, we utilized *myo-3p*:GFP-overexpressing worms, which express a GFP under the control of the myosin-3 promoter, to quantify mitochondria content by BioSorter analysis in the muscle of DLD-1–deficient worms. An inverse relationship was observed between the amount of DLD-1 protein and *C*. *elegans* body wall muscle mitochondrial mass. Specifically, *myo-3p*:GFP worms grown on 1:20 dilution of *dld-1(RNAi)* that had 38% reduced DLD-1 protein expression had no significant change in muscle mitochondrial content relative to control worms. In contrast, worms grown on full-dose *dld-1(RNAi)* that had 71% reduced DLD-1 protein expression had a 21% increased mitochondrial content relative to control worms (Figure 5E, *P* < 0.005). Thus, severe DLD-1 deficiency in *C*. *elegans* leads to a mitochondrial proliferation adaptive response in their skeletal muscle.

Overall, mechanistic biochemical studies demonstrated that full-dose RNAi depletion of DLD-1 in *C*. *elegans* induced significant mitochondrial stress, increased pyruvate levels and ratio of pyruvate/lactate consistent with increased levels of NAD^+^ from its diminished conversion to NADH by E3-containing mitochondrial enzymes, reduced ATP levels, and induced muscle mitochondrial proliferation as an adaptive response to their complex mitochondrial dysfunction. This cellular pathophysiology disrupted overall animal health in DLD-1–deficient worms, as evidenced by their altered survival, decreased adult linear growth, reduced fecundity, and impaired neuromuscular function.

### Empiric DLD deficiency therapies, DCA and thiamine, rescue life span in short-lived, DLD-1–deficient C. elegans.

**Figure 6:**
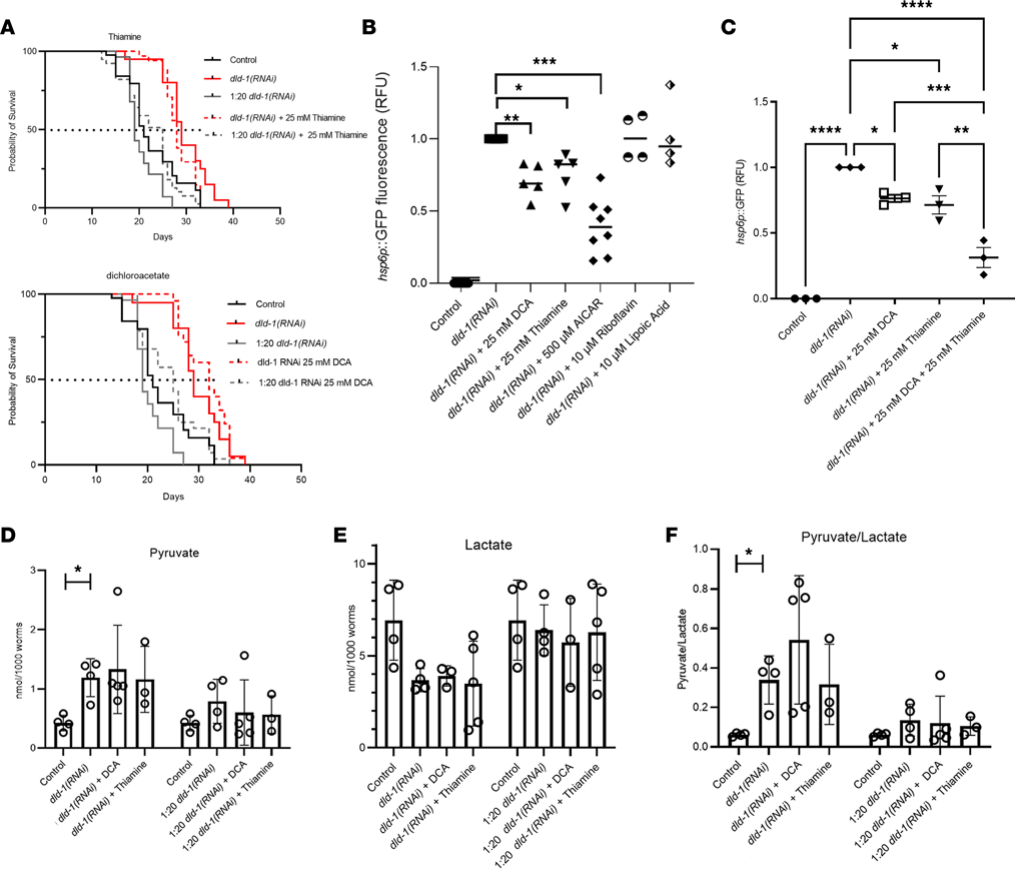
DCA and thiamine demonstrated significant benefit on mitochondrial stress and survival but not on pyruvate or lactate levels. (**A**) Survival analysis compared 1:20 and full-dose *dld‑1*(*RNAi*) worms with those treated from L4 in 3 replicates with 25 mM DCA or 25 mM thiamine. Log-rank (Mantel-Cox) tests indicated both treatments significantly rescued the decreased survival of the partial knockdown (thiamine *P* < 0.05, HR = 0.49; DCA *P* < 0.001, HR = 0.48). Neither treatment affected the extended life span of full-dose *dld‑1(RNAi)*. (**B**) Mitochondrial stress. Only 25 mM DCA (**P < 0.01), 25 mM thiamine (*P < 0.05), and 500 μM AICAR (***P < 0.001) significantly reduced UPR^mt^ assayed by *hsp6p*:GFP fluorescence relative to *dld-1*(*RNAi*) knockdown worms analyzed by BioSorter. Significance was analyzed by 1-way ANOVA followed by Dunnett’s multiple comparisons. (**C**) DCA and thiamine significantly reduced UPR^mt^ in 4 additional biological replicates; mean ± SEM shown. Significance was obtained by 1-way ANOVA followed by Tukey’s multiple tests (DCA alone, *P < 0.05; thiamine alone, *P < 0.05). Combination of DCA and thiamine resulted in significantly in the greatest reduction (****P < 0.0001), which was significantly greater than observed with either treatment alone: thiamine added to DCA (***P < 0.001) and DCA added to treatment with thiamine (**P < 0.01). (**D**) Pyruvate levels were determined in 4 replicates of 7 cohorts of 1,000 worms grown in parallel: N2 controls, N2 fed *dld-1(RNAi)*, or 1:20 *dld-1(RNAi)* with either DCA or thiamine. Increased pyruvate was observed in *dld-1(RNAi)* knockdown worms (*P < 0.05), but neither DCA nor thiamine treatments resulted in a significant decrease. With the 1:20 *dld-1(RNAi)*, the increase in pyruvate level was not significant and not significantly changed by DCA or thiamine. (**E**) Lactate levels decreased with *dld-1(RNAi)* treatment and were insignificantly changed with either drug. (**F**) Pyruvate/lactate ratio was significantly increased by *dld-1(RNAi)* knockdown but insignificantly reduced by either drug. Analyses for **D**–**F** were a Student’s 2-tailed *t* test for the effect of the *dld-1(RNAi)* knockdown and 1-way ANOVA for the effect of the drugs.

As animal life span was significantly increased by full-dose and significantly decreased by partial 1:20 DLD-1 knockdown (Figure 3C), we investigated whether the variably altered life span of DLD-1–deficient *C*. *elegans* animals could be normalized toward that of wild-type worms by therapies that are empirically used in human patients with DLD deficiency (7). Specific therapies studied included, among others shown in Supplemental Table 1, the PDHc activator DCA; α-ketoacid dehydrogenase complex cofactors thiamine (vitamin B_1_), riboflavin (vitamin B_2_) (7, 35), or lipoic acid (36); NAD^+^ precursors nicotinic acid (37) and nicotinamide; the metabolic modifier l-carnitine (38); and 5-aminoimidazole-4-carboxamide-1-β-d-ribofuranoside (AICAR). Worms were grown from L1 stage on control, *dld‑1(RNAi)* only, or 1:20 *dld-1(RNAi)* dilution bacteria along with individual drug treatments to achieve comparative analysis of life span and growth effects in synchronous adult worm populations. L4 larval stage animals were then transferred to nematode growth media (NGM) plates containing the specified drug therapies for the duration of the experiment. In 3 biological replicates, DCA (25 mM) and thiamine (25 mM) treatments each significantly increased the life span of short-lived worms grown on 1:20 *dld-1(RNAi)* dilution bacteria (*P* < 0.05 for thiamine; *P* < 0.001 for DCA) compared with buffer-only exposed 1:20 *dld-1(RNAi)* worms, with full rescue of the median life span of the partially DLD-deficient worms to that of worms grown on control RNAi (Figure 6A). However, neither DCA nor thiamine treatment significantly normalized the life span of the long-lived worms grown on full-dose *dld-1(RNAi)* (Figure 6A). Riboflavin (10 μM), l-carnitine (100 μM), AICAR (500 μM), and lipoic acid (10 μM) did not significantly normalize animal life span of DLD-deficient worms grown at either full-dose or 1:20 *dld-1(RNAi)* dilution (Supplemental Figure 5, A–C). Interestingly, lipoic acid (10 μM) treatment actually further decreased median life span by 38% (*P* < 0.001) of short-lived worms grown on 1:20 *dld‑1(RNAi)* dilution as compared with buffer-only controls (Supplemental Figure 5C). Overall, full rescue of short-lived animal survival by treatment with either DCA or thiamine in 1:20 *dld-1(RNAi)* partially DLD-deficient animals provided what may be the first objective evidence to suggest these treatments indeed hold efficacy to restore the pathophysiologic effects of DLD disease. However, none of the 5 empiric studies modeled normalized the extended life span of severely DLD-deficient worms grown on full-dose *dld-1(RNAi)*.

### Empiric DLD therapies variably restore abnormal mitochondrial physiology in DLD-1–deficient C. elegans.

Since depletion of DLD-1 in *C*. *elegans* disrupted multiple aspects of mitochondrial physiology, we evaluated whether empiric therapies used in human DLD disease objectively normalized mitochondrial physiology in DLD-1–deficient worms. Effects of DCA, thiamine, riboflavin, lipoic acid, AICAR, and l-carnitine at the same concentrations studied on animal life span were evaluated on mitochondrial stress, mitochondrial content, and relative mitochondrial membrane potential of young adult worm populations grown from the L1 larval stage on control, full-dose *dld-1(RNAi)* only, or 1:20 *dld-1(RNAi)* dilution until time of analysis. Riboflavin, lipoic acid, nicotinic acid, nicotinamide, and l-carnitine had no effect on the increased UPR^mt^ of worms exposed to full-dose or 1:20 dilution of *dld‑1(RNAi)* (Figure 6B and Supplemental Table 1). However, the markedly increased UPR^mt^ induction caused by DLD-1 deficiency in *C*. *elegans* was significantly reduced by 30% with DCA treatment (*P* < 0.01) and by 25% with thiamine treatment (*P* < 0.05), based on *hsp-6p*:GFP fluorescence quantitation in *hsp-6p*:GFP worms grown on full-dose *dld‑1(RNAi)* (Figure 6B). To determine if there was a dose response to DCA and thiamine, the effect of concentrations from 0.1 to 25 mM of each compound was tested on UPR^mt^. For both DCA and thiamine a trend was seen toward increased protection from UPR^mt^ induction, which became highly significant at 25 mM of either compound (Supplemental Figure 6, A and B). To determine if a combination would be more effective, 4 biological replicate experiments were performed with 25 mM of each compound, demonstrating that the DCA and thiamine combination was significantly more effective than either treatment alone (*P* < 0.01, Figure 6C).

Interestingly, the increased pyruvate level and pyruvate/lactate ratio that occurred upon full-dose *dld-1(RNAi)* in worms relative to untreated N2 (*P* < 0.05) were not significantly prevented with either thiamine treatment or DCA (Figure 6, D–F). Thus, beneficial effects of DCA and thiamine on reducing mitochondrial stress in DLD-1–deficient worms were not mediated by normalization of their increased pyruvate level or of their increased pyruvate/lactate ratio that is likely reflective of their increased NAD^+^/NADH redox balance.

## Discussion

Autosomal recessive pathogenic variants in human *DLD*, which encodes DLD that functions as the common E3 subunit of 4 mitochondrial matrix enzymes, lead to a debilitating primary mitochondrial disease. No viable animal models exist in which to pursue deeper investigation of DLD disease pathogenesis or the objective safety and utility of commonly prescribed empiric therapies. In a mouse model, deletion of *Dld* resulted in embryonic lethality of the homozygote and no identifiable phenotype of the heterozygote (18). In a zebrafish morpholino model, gross morphological changes at 6 days postfertilization were apparent with decreased heart rate, while a KGDHc-specific homozygous knockout mutant of the dihydrolipoylsuccinyl transferase was also embryonic lethal (39). Here, we used a graded RNAi feeding gene knockdown approach to model partial and severe DLD deficiency in *C*. *elegans* (23). RNAi inhibition provides a viable model in which effects on whole-animal survival and physiology can be quantified of either partial or severe DLD-1 protein and E3 enzymatic deficiency resembling those observed, respectively, in the heterozygous asymptomatic carrier or homozygous disease state in humans (23). Indeed, we demonstrated that DLD-1 depletion significantly altered animal survival (Figure 3C) and impacted diverse aspects of animal health, including decreased brood size, shortened adult length, and impaired neuromuscular function. Further, DLD-1–deficient worms displayed the classic biochemical hallmark of an increased pyruvate/lactate ratio that occurs in many etiologies of PDHc deficiency, which are frequently associated with increased absolute levels of both pyruvate and lactate. A greater proportional increase in pyruvate greater than that in lactate gives rise to the diagnostic decrease in the lactate/pyruvate ratio (or an increase in the pyruvate/lactate ratio) (40–47), in contrast to the increased lactate/pyruvate ratio that occurs due to increased NADH/NAD^+^ redox ratio in primary RC disease (7, 44, 48). Indeed, the worm tissue homogenate showed a trend toward increased pyruvate levels and decreased lactate levels, which may relate to the analysis being performed on the homogenate of a population of entire organisms rather than the extracellular environment that is analyzed in standard human clinical blood-based analyses.

In addition, our detailed dissection of the complex mitochondrial pathophysiology of DLD-1–deficient *C*. *elegans* provides what may be the first evidence to link DLD deficiency with a broader array of mitochondrial pathophysiology including ATP deficiency and likely adaptive muscle mitochondrial proliferation, without significantly altered RC enzyme activities or integrated mitochondrial membrane potential, suggestive of systemic metabolic changes and a more greatly oxidative environment than in wild-type animals. Dramatically, DLD-1–deficient worms manifest a 33-fold increase in the mitochondrial unfolded stress response as quantified by the *hsp6p*-induced expression of GFP. Screening of empiric therapies yielded no benefits of riboflavin, lipoic acid, and l-carnitine on DLD-1–deficient worm survival or mitochondrial physiology. Importantly, significant improvement of animal survival in 1:20 *dld-1(RNAi)* dilution worms with partial DLD-1 deficiency, as well as significant reduction of mitochondrial stress response in both partial and full-dose *dld-1(RNAi)* worms, was seen with both DCA and thiamine. Importantly, the combination of both DCA and thiamine demonstrated a greater effect than either treatment alone. However, these beneficial effects on mitochondrial stress of DCA and thiamine were not mediated by significant normalization of the increased pyruvate level and pyruvate/lactate ratio that occurred in full-dose *dld-1(RNAi)* worms. Intriguingly, a case report previously demonstrated this combinatorial therapy, along with carnitine, led to clinical improvement in a pediatric DLD disease patient (49).

The differential effects of variable degrees of DLD depletion on animal survival are intriguing. Specifically, our work corroborated a previous report demonstrating that partial DLD-1 deficiency reduced median survival in *C*. *elegans*, whereas more severe DLD-1 deficiency actually extended animal survival (23). While the cause of the biphasic impact on animal survival of variably graded DLD-1 knockdown has yet to be identified, rich literature demonstrates the pivotal role that modulating DLD activity may play in regulating the survival of cancer cells. Inhibition of DLD in melanoma cells resulted in an increased ratio of NAD^+^ to NADH, with a concomitant reduction in TCA cycle intermediates, increased ROS production, and enhanced autophagic cell death (50). Further, knockdown or reduction of DLD activity through ultraviolet-A irradiation reduced tumor size and progression in a mouse model of melanoma (50). Following the identification of lipoic acid derivatives as specific inhibitors of the ketoacid dehydrogenases and demonstration of their efficacy as potential therapeutics in both cell and animal models (51), a large number of studies have focused on the potential of PDHc and KGDHc inhibitors and regulators as cancer chemotherapeutic agents (52). Conversely, oncogenic transformation is facilitated by the enhanced expression of PDHc kinases, resulting in increased glycolysis over oxidative phosphorylation for ATP production. To counteract this effect, inhibitors of PDHc kinases have been developed (53). Given their reduced DLD function, as anticipated, no cancer predisposition has been reported in human patients with *DLD* disease (54).

While the relative contributions of different biochemical pathways to cellular dysfunction and clinical phenotypes of *DLD* disease have not been fully clarified, disease pathogenesis has most often been attributed to a PDHc deficiency. *C*. *elegans* phenotypes observed here upon RNAi-mediated DLD-1 depletion provide evidence for broader mitochondrial dysfunction, leading to an overall decrease in ATP levels, similarly as has previously been reported in DLD patient fibroblast cell lines (55). The enhancement of the combined efficacy of DCA and thiamine treatment over the effect of either alone supports this broader view. The ATP depletion could not be attributed in our DLD-deficient *C*. *elegans* models to significantly reduced individual RC enzyme activities or integrated RC capacity as evaluated by in vivo mitochondrial membrane potential analysis. Rather, ATP depletion in DLD deficiency may result from a deficiency in the TCA cycle, as both PDHc and KGDHc are expected to be deficient due to their lack of E3 subunits. It is plausible that this dysfunction limits generation of reducing equivalents to enter the RC or that accumulation of α-ketoglutarate inhibits RC complex V function, as has been previously described (56). Interestingly, increased α-ketoglutarate was also found to increase animal life span in *C*. *elegans*, which could potentially contribute to the prolonged survival seen with full-dose RNAi depletion of DLD-1 (56). Future investigations of intermediary cellular metabolism and metabolic flux alterations in the DLD-1–deficient *C*. *elegans* model may provide additional insights into the cause of decreased ATP that results from primary depletion of DLD.

Multiple adaptive responses commonly occur in a primary mitochondrial disorder. Indeed, mitochondrial proliferation has been reported in muscle biopsies of patients with DLD (35), a finding that our model recapitulated with significantly increased mitochondrial content in DLD-1–depleted *C*. *elegans* (Figure 5E). Furthermore, the UPR^mt^ is a highly conserved stress response pathway in which nuclear gene expression of antioxidant and heat shock proteins is induced in attempts to compensate for defective mitochondrial function(s) and restore mitochondrial homeostasis. Interestingly, UPR^mt^ induction has been linked to increased animal longevity (57). The substantial induction of the UPR^mt^ stress response in DLD-1–deficient worms is consistent with, and may also potentially contribute to, the prolonged survival of full-dose *dld-1(RNAi)* inhibited worms that manifest 71% depletion of DLD-1 expression and 66% depletion of E3 enzymatic activity. Clear demonstration of adaptive mitochondrial content and mitochondrial stress responses in the *C*. *elegans* model of DLD deficiency provide strong evidence to suggest the pathophysiology of *DLD* disease exceeds that attributable only to deficiency of the single enzyme complex, PDHc.

Overall, these preclinical data demonstrate that graded RNAi inhibition of DLD-1 expression and activity in *C*. *elegans* provides a viable and robust invertebrate animal model of *DLD* disease, which displays the classic biochemical hallmark of increased pyruvate levels commonly occurring in any case of PDHc deficiency. DLD-1–deficient animals had severely altered animal survival and impairment of diverse aspects of animal health, including decreased brood size, shortened adult length, and impaired neuromuscular function. Detailed characterization of the complex mitochondrial pathophysiology of DLD-1–deficient *C*. *elegans* provided evidence to link DLD deficiency with a broader constellation of mitochondrial pathophysiology, including a dramatically increased mitochondrial stress response, an adaptive mitochondrial proliferation, and marked ATP deficiency, despite normal RC enzyme activities and integrated mitochondrial RC capacity at the level of mitochondrial membrane potential. Most importantly, preclinical therapeutic modeling of 5 standard empiric therapies used in *DLD* disease demonstrated that only 2, DCA and thiamine, showed objective benefit in the treatment of DLD-1 disease, findings that warrant further evaluation in rigorous human clinical treatment trials. This combination therapy was attempted in 1 early case report where the thiamine administration proved beneficial but the DCA dose was discontinued (58) and a more recent report where PDH deficiency was stabilized in patients (59). More recent characterization of the complex pharmacodynamics of DCA may lead to development of a more effective treatment (60). We postulate that successful treatment of *DLD* disease may well require a similar combinatorial therapy approach, as multiple mitochondrial enzyme complexes are impacted by DLD deficiency and our work demonstrates that global mitochondrial metabolism is impaired with ATP deficiency, increased pyruvate level and pyruvate/lactate ratio, along with a greatly enhanced mitochondrial stress response (61). Future work using the *C*. *elegans* *dld-1(RNAi)* inhibition model can provide preclinical evaluation of additional drug therapy leads that may ultimately enable identification of safe and potent lead candidates to translate back in clinical trials to evaluate their therapeutic benefit in humans with *DLD* disease.

## Methods

### C.

*elegans strains and maintenance*. *C*. *elegans* WT N2 Bristol worms, SJ4103 *zcIs14[myo-3p:GFP(mit)]*, and JJ2586 *cox-4(zu476[cox-4:eGFP:3xFLAG]*) were obtained from the Caenorhabditis Genetics Center (http://www.cbs.umn.edu/CGC/). A strain expressing GFP under the control of the HSP6 promoter, st*zcIs13*[*hsp-6_p_:GFP*] (32), was a gift from Cole Haynes (University of Massachusetts Chan Medical School, Worcester, Massachusetts, USA). Animals were maintained at 20°C on NGM plates spread with *E*. *coli* OP50 (obtained in-house). Gene knockdown experiments were performed by feeding bacteria transfected with plasmids to inducibly express RNAi with either L4440 (control RNAi), or LLC1.3 [*dld-1(RNAi)*]. Bacterial clones were obtained from the Ahringer RNAi collection (Source BioScience) (62) and sequence verified. IPTG (0.2 mM) was used to induce feeding RNAi expression.

### C. elegans brood size analysis.

Worms were synchronized by picking 10 to 15 gravid adult N2 worms onto NGM plates containing 0.2 mM IPTG, ampicillin, and tetracycline spread with *dld-1(RNAi)* or empty vector control bacteria and allowed to lay eggs for 2 to 2.5 hours. A single L4 larva from the egg laying was placed on a 3.5 cm NGM plate spread with the appropriate feeding RNAi clone containing 0.2 mM IPTG and allowed to lay eggs for 24 hours before being transferred to a fresh 3.5 cm NGM plate spread with the same RNAi clone and 0.2 mM IPTG. Worms were transferred to fresh plates spread with the same RNAi clone and 0.2 mM IPTG for 5 days, and total progeny were counted from each plate.

### Immunoblotting.

*C*. *elegans* lysates were prepared from approximately 500 young adult worms that were briefly washed with S basal media (5.85 g NaCl, 1 g K_2_HPO_4_, 6 g KH_2_PO_4_, and 5 mg cholesterol, in 1 L H_2_O) before resuspension in 250 μL of RIPA buffer (50 mM Tris-HCl, pH 8.0, 150 mM NaCl, 1.0% NP-40, 0.5% sodium deoxycholate, 1.0 mM EDTA, 0.1% SDS) containing a 1:100 dilution of protease inhibitor cocktail (MilliporeSigma). Worms were lysed using a mechanical hand homogenizer and pestle on ice. The resulting lysates were maintained at constant agitation for 30 minutes at 4°C. Lysates were subsequently centrifuged for 20 minutes at 4°C at 16,000*g*, and the supernatant was removed. Protein concentrations were determined using the Pierce BCA protein assay kit. Whole worm lysates (30 μg of protein) were loaded onto duplicate 4%–15% Tris-glycine gel, and blots were probed with anti-LAD monoclonal antibody at a 1:7,500 dilution (Abcam, catalog ab133551) to detect the DLD-1 protein and, as a loading control for *C*. *elegans* protein, were probed with anti–β-tubulin (MilliporeSigma, catalog T2200) at a 1:1,000 dilution. Gel fluorescence was imaged with an Odyssey CLx using excitation at 685 nm and 785 nm and quantified via ImageJ.

### Neuromuscular activity analysis by chemotaxis assay in C. elegans.

Chemotaxis analysis was performed as has been previously described (28). In brief, approximately 30 worms in each of 3 technical replicates were collected and washed with S basal media. Worms were placed 5 cm away from a spot containing 2 μL of 10% isoamyl alcohol dissolved in 100% ethanol and 1 μL of 10% sodium azide. After 1 hour, the distance traveled by each worm was manually marked and analyzed via ImageJ.

### Quantification of animal length in C. elegans.

Synchronized populations of worms were grown on control (L4440), *dld-1(RNAi)* (LLC1.3), or 1:20 *dld-1(RNAi)*. Each population was monitored for development at the L4 stage and days 1, 5, and 10 of adulthood. At the L4 stage, worms from each population were transferred onto plates containing 50 μM fluorodeoxyuridine to prevent the birth of progeny for analysis at adult days 1, 5, and 10. On the appropriate days, L4 larvae to day 10 adults were collected from the plates and washed in 4 to 5 mL S basal in a 50 mL conical tube (obtained from Children’s Hospital of Philadelphia). Flow cytometry analyses of animal length were performed using a BioSorter (Union Biometrica), which measures the relative axial length of an object by an axial light loss detector, where time of flight (TOF) indicates animal length (63). TOF measurements were collected for approximately 100–300 worms per sample, with 4 biological replicates tested per condition.

### Quantification of hsp-6p:GFP induction in C. elegans.

Synchronized *hsp-6p:*GFP worm populations were grown from birth on control or *dld-1(RNAi)* bacteria to induce depletion of DLD-1 protein. Day 2 adult worms were then washed in 4 to 5 mL of S basal in a 50 mL conical tube and pelleted by gravity. Supernatant was removed and worms were resuspended in a final volume of 10 mL S basal. To quantify relative *hsp-6p:*GFP induction, Biosorter analysis was performed using λ_excitation_ = 488 nm and λ_emission_ = 510 nm and the fluorescence intensity integrated during the worms’ transit. Experiments were conducted with approximately 300 worms per sample, with 3 biological replicates performed for each condition tested. To assess the efficacy of therapies on *hsp-6p*:GFP expression, treatment was started from egg hatching with these tested compounds: DCA, thiamine, riboflavin, AICAR, and lipoic acid. Dose-response curves were generated for DCA and thiamine by varying the concentration of drug from 0.1 to 25 mM in 4 biological replicates. The effect of a combination of DCA and thiamine at the most effective dose of 25 mM was included in the 4 replicates.

### Quantification of C. elegans in vivo mitochondrial membrane potential.

Living worms’ mitochondrial membrane potential was relatively quantified by fluorescence analysis of the red TMRE fluorescence normalized to the green fluorescence of the mitochondrial reporter COX4:GFP in whole worms imaged with a CellInsight CX5 high-content scanner as adapted (64). Specifically, approximately 150 to 200 COX4:GFP worms were grown from birth on either control, 1:20 dilution of *dld-1(RNAi)* and L4440 control bacteria, or full-dose *dld-1(RNAi)* only to the young adult stage. Young adult worms were then washed with 6 mL S basal and incubated in S basal for 30 minutes to allow gut clearance of bacteria prior to TMRE staining. The S basal was then removed and replaced with 1 μM TMRE in 300 μL of S basal followed by a 6-hour incubation while worms were gently rotated at 20°C. The TMRE solution was removed and worms were washed with 1 mL of fresh S basal 4 times to remove residual dye prior to a 30-minute incubation at room temperature in S basal to allow for gut clearance of residual fluorescent dye. Cleared worms were collected in 5 mL of S basal and studied by CX5 HCS image analysis. Approximately 30 worms in 60 μL of S basal were transferred to a single well in a 384-well optical bottom CellInsight CX5 HCS plate, and the worms were paralyzed by adding 40 μL of 20 mM levamisole. Images were obtained from both the GFP (COX4, a mitochondrial content marker) and red fluorescence protein (RFP) (TMRE, a mitochondrial membrane potential marker) channels with 4× original magnification (Supplemental Figure 4). The RFP/GFP channel ratios for each worm were determined, and the ratio was normalized to the CX5 object area. As increased membrane potential is revealed by increased mitochondrial matrix uptake of the positively charged TMRE dye, an increase in this ratio corresponds to an increase in membrane potential.

### Quantification of relative mitochondrial content in C. elegans body wall muscle.

Mitochondrial content of living worms was relatively quantified by Biosorter in *myo-3p*:GFP(mit) animals, which express a GFP protein with a mitochondrial leader sequence under the control of the *myo-3* promoter in the muscle of the *C*. *elegans* body wall (65). In brief, *myo-3p*:GFP(mit) animals were grown from birth to the young adult stage on bacteria-fed control (L4440), 1:20 dilution of *dld-1(RNAi)* and L4440, or only *dld-1*(*RNAi)*. Young adult worms were collected in 5 mL of S basal and studied by BioSorter analysis utilizing λ_excitation_ = 488 nm and λ_emission_ = 510 nm for fluorescence and normalized for size, determined as the product of the TOF, and extinction was determined for each worm.

### C. elegans life span analysis.

Life span analyses of *C*. *elegans* were performed at ambient temperatures around 20°C. Gravid adult worms grown on solid NGM media containing OP50 were bleached to obtain eggs to achieve synchronous worm populations. Isolated eggs were then transferred to plates containing control or *dld‑1(RNAi)* clones and grown to the L4 larvae stage. Approximately 90 L4 larvae were plated onto 3 plates containing 50 μM fluorodeoxyuridine. Worm survival was assessed using standard methods as previously described and analyzed in GraphPad Prism 7.04. To assess the efficacy of therapies on life span, treatment was started from egg hatching with these tested compounds: DCA, thiamine, riboflavin, lipoic acid, and l-carnitine.

### Sample preparation for biochemical analyses.

Worms were grown from hatching at 20°C on NGM plates spread with *E*. *coli* OP50 and were collected with about 1,000 worms per tube. After washing 3 times with S basal, the buffer was removed, and the worms were immediately flash-frozen in liquid N_2_ and then stored at –80°C until used for analyses. For ATP, lactate, and pyruvate assays, frozen worms were homogenized in ice-cold 0.5 M perchloric acid (PCA) by grinding with a motorized pestle, 1 second of sonication, and several freeze/thaw cycles in liquid N_2_/ambient temperature water. After centrifuging at 4°C at 16,000*g* for 15 minutes, the supernatant was collected and neutralized by ice-cold 1 M potassium carbonate. The supernatant after centrifuging at 4°C at 16,000*g* for 10 minutes was used. For E3 and RC enzyme activity assays, frozen worms were treated with proteinase K (1 mg/mL) for 10 minutes in a mitochondrial isolation buffer (250 mM sucrose, 20 mM Tris-HCl, 3 mM EDTA, pH 7.4) on ice, and then, 5 mM PMSF was added to inactivate the proteinase. Worms were washed twice and homogenized in the buffer with a motorized pestle, followed by 1 freeze/thaw cycle with liquid N_2_. Mitochondria-enriched fractions were obtained by differential centrifugation. Assays were performed at 37°C for lactate and pyruvate colorimetric assays, or 30°C for E3 and RC enzyme activity spectrophotometric assays in 170 μL final volume using a Tecan Infinite 200 PRO plate reader.

### Lactate assay.

We have developed a highly sensitive lactate oxidase–based (LOX-based) colorimetric assay for lactate analysis using a dye (61), (Carboxymethylaminocarbonyl)-4,4′-bis(dimethylamino)diphenylamine sodium salt (DA-64), which has been shown to be a highly sensitive indicator for the detection of hydrogen peroxide (H_2_O_2_) (66). LOX catalyzes the reaction l‑lactate + O_2_ → pyruvate + H_2_O_2_. Five μL of neutralized PCA extract was added to 155 μL of lactate assay reaction mixture (0.2 mM DA-64, 1 mM EDTA, 0.1% Triton X-100, and 5 U/mL HRP in 100 mM HEPES, pH 7.4), mixed thoroughly, and then incubated at 37°C for 3 minutes. Afterward 10 μL of LOX (freshly prepared at 2 U/mL) was added to each well, and the absorbance was measured every 20 seconds at 727 nm for 15 minutes. Lactate concentrations in samples were calculated using standard curves generated with sodium l‑lactate.

### Pyruvate assay.

We have similarly developed a pyruvate oxidase–based (POX-based) colorimetric determination of the equimolar H_2_O_2_ generated during the oxidative decarboxylation of pyruvate (61) by using the dye DA-64 (66). POX catalyzes the conversion of pyruvate to acetylphosphate and H_2_O_2_ as shown: pyruvate + HPO_4_^2–^ + O_2_ → acetylphosphate + CO_2_ + H_2_O_2_. In this reaction, the cofactors FAD, thiamine pyrophosphate (TPP), and Mg^2+^ are required. Twenty-five or 50 μL of neutralized PCA extract was added to 110–135 μL of pyruvate assay reaction mixture (10 μM FAD, 0.2 mM TPP, 10 mM MgCl_2_, 0.2 mM DA-64, 1 mM EDTA, 0.1% Triton X-100, and 5 U/mL HRP in 100 mM KH_2_PO_4_, pH 6.0), mixed thoroughly, and then incubated at 37°C for 3 minutes. Afterward 10 μL of POX (freshly prepared at 2 U/mL) was added to each well and the absorbance at 727 nm was measured every 20 seconds for 15 minutes. Pyruvate concentrations in samples were calculated using standard curves with sodium pyruvate.

### ATP assay.

Separation of ATP was performed using a YMC-Pack ODS-A column (5 μm, 4.6 × 250 mm) preceded by a guard column at 50°C. Using a 0.4 mL/min flow rate, a 10-minute linear gradient from 100% mobile phase A (0.1 M sodium phosphate buffer, pH 6.0) to 20% mobile phase B (0.1 M sodium phosphate buffer, pH 6.0, containing 25% methanol v/v) cleanly separated ATP. The column was washed after each separation by increasing mobile phase B to 100% for 5 minutes. UV absorbance at 260 nm was recorded with a Shimadzu SPD-M20A. Pertinent peak areas were integrated by the LabSolution software from Shimadzu, quantified using standard curves, and normalized per 1,000 worms.

### E3 and RC complex enzyme activity assays.

Mitochondrial RC complex I and complex II enzyme activities were determined by the reduction of 2,6-dichlorobenzenone-indophenol sodium salt (2,6-DCPIP) at 600 nm (ε_600_ = 21/mM/cm). The assay buffer for complex I assay contained 25 mM KH_2_PO_4_, pH 7.4, 5 mM MgCl_2_, 3 mg/mL BSA, 25 μM ubiquinone Q1, and 5 μM antimycin A and mitochondrial worm extract. The reaction was started by the addition of 100 μM NADH in the presence and absence of 5 μM rotenone; the rates were calculated after the subtracting the rotenone-insensitive activities. The assay buffer for complex II assays contained 25 mM KH_2_PO_4_ pH 7.4, 5 mM MgCl_2_, 3 mg/mL BSA, 25 μM ubiquinone Q1, 5 μM antimycin A, 5 μM rotenone, and mitochondria-enriched worm extract. The reaction was started with 20 mM succinate. Complex IV activity was measured by following the oxidation of reduced cytochrome *c* at 550 nm (ε_550_ = 21/mM/cm). The assay buffer contained 25 mM KH_2_PO_4_ pH 7.4, 5 mM MgCl_2_, 0.015% *n*‑dodecyl-β-d-maltoside, 5 μM antimycin A, 5 μM rotenone, and mitochondria-enriched worm extract. The reaction was started with 15 μM reduced cytochrome *c*. The rates were calculated as a first order rate constant. Dihydrolipoyl dehydrogenase (E3) activity was determined by the reduction of 2,6-DCPIP at 600 nm (ε_600_ = 21/mM/cm). The assay buffer for E3 assays contained 50 mM KH_2_PO_4_, pH 7.0, 1.25 mM EDTA, and 400 μM lipoamide and mitochondria-enriched worm extract; the reaction was started with 100 μM NADH. The rates were calculated after the subtraction of lipoamide-insensitive activities. Because of the diaphorase activity of E3 in the presence of DCPIP, this assay is only valid for saturating lipoamide concentrations. The specific activity was normalized by protein concentration determined by Bradford assay (67).

### Statistics.

Unless otherwise noted, differences between control worms and worms treated with *dld-1(RNAi)* were determined by averaging measurements from worms grown in parallel biological replicate experiments and the means and SDs presented. Statistical significance for comparison of control, 1:20 *dld-1(RNAi)*, and full-dose *dld-1(RNAi)* worms was determined by 2-sided 1-way ANOVA followed by Tukey’s multiple comparisons. For analyses of large numbers of worms conducted with the Biosorter, SEM is presented. When cohorts of both 1:20 *dld-1(RNAi)* and undiluted *dld-1(RNAi)* were analyzed at several different days of adulthood, 2-way ANOVAs were used. Significance levels were defined for *P* values less than 0.05, 0.01, 0.001, and 0.0001. Survival analyses were performed with a log-rank (Mantel-Cox) test. All data were analyzed with GraphPad Prism 9.4.0.

### Study approval.

No vertebrate animals, human patients, or human specimens were used in the reported studies.

## Author contributions

MJF conceived of and designed the study. CNB, PK, and SG performed fluorescence analyses of mitochondrial physiology in *C*. *elegans*. CNB and PK performed *C*. *elegans* life span and growth analyses. PK performed the combination drug therapy experiments. ENO performed RC, ATP, E3, and lactate/pyruvate studies and wrote the corresponding Methods sections. NM contributed the bioinformatics analysis and RX assisted with the statistical methods and analyses. ML assisted with the design and preparation of the figures. CNB, VEA, and MJF wrote the manuscript. All authors approved of the final version.

## Supplementary Material

Supplemental data

## Version 1 10/24/2022

Electronic publication
