# Supplementary material for: Dichloroacetate and thiamine improve survival and mitochondrial stress in a C. elegans model of dihydrolipoamide dehydrogenase deficiency
Source: JCI Insight. 2022 Oct 24;7(20):e156222. doi: 10.1172/jci.insight.156222 (PMC9714793; doi:10.1172/jci.insight.156222)

Supplemental Figure 1

```
                          Mitochondrial Targeting Sequence         FAD-binding domain
H.sapiens      MQSWSRVYCSLAKRGHFNRISHGLQGLSAVPLRTYADQPIDADVTVIGSGPGGYVAAIKA 60
C.elegans      MSLSRTTQLPFAKRQFF-----------QVLARNY-SNTQDADLVVIGGGPGGYVAAIKA 48
               *.    .   :*** .*            *  *.* .:  ***:.***.***********

H.sapiens      AQLGFKTVCIEKNETLGGTCLNVGCIPSKALLNNSHYYHMAHGTDFASRGIEMSEVRLNL 120
C.elegans      AQLGMKTVCVEKNATLGGTCLNVGCIPSKALLNNSHYLHMAQ-HDFAARGIDCT-ASLNL 106
               ****:****:*** *********************** ***:  ***:***: : . ***

H.sapiens      DKMMEQKSTAVKALTGGIAHLFKQNKVVHVNGYGKITGKNQVTATKADGGTQVIDTKNIL 180
C.elegans      PKMMEAKSNSVKQLTGGIKQLFKANKVGHVEGFATIVGPNTVQAKKNDGSVETINARNIL 166
                **** **.:** ***** :*** *** **:*:..*.* * * *.* **..:.*:::***
                                                     NAD-binding domain
H.sapiens      IATGSEVTPFPGITIDEDTIVSSTGALSLKKVPEKMVVIGAGVIGVELGSVWQRLGADVT 240
C.elegans      IASGSEVTPFPGITIDEKQIVSSTGALSLGQVPKKMVVIGAGVIGLELGSVWQRLGAEVT 226
               **:**************. ********** :**:***********:***********:**

H.sapiens      AVEFLGHVGGVGIDMEISKNFQRILQKQGFKFKLNTKVTGATKKSDGKIDVSIEAASGGK 300
C.elegans      AVEFLGHVGGMGIDGEVSKNFQRSLTKQGFKFLLNTKVMGASQN-GSTITVEVEGAKDGK 285
               **********:*** *:****** * ****** ***** **::: ...* *.:*.*..**
                                                Central domain
H.sapiens      AEVITCDVLLVCIGRRPFTKNLGLEELGIELDPRGRIPVNTRFQTKIPNIYAIGDVVAGP 360
C.elegans      KQTLECDTLLVSVGRRPYTEGLGLSNVQIDLDNRGRVPVNERFQTKVPSIFAIGDVIEGP 345
                :.: **.***.:****:*:.***.:: *:** ***:*** *****:*.*:*****: **

H.sapiens      MLAHKAEDEGIICVEGMAGGAVHIDYNCVPSVIYTHPEVAWVGKSEEQLKEEGIEYKVGK 420
C.elegans      MLAHKAEDEGILCVEGIAGGPVHIDYNCVPSVVYTHPEVAWVGKAEEQLKQEGVAYKIGK 405
               ***********:****:*** ***********:***********:*****:**: **:**
                                          Interface domain
H.sapiens      FPFAANSRAKTNADTDGMVKILGQKSTDRVLGAHILGPGAGEMVNEAALALEYGASCEDI 480
C.elegans      FPFVANSRAKTNNDQEGFVKVLADKQTDRMLGVHIIGPNAGEMIAEATLAMEYGASAEDV 465
               ***.******** * :*:**:*.:*.***:**.**:**.****: **:**:*****.**:

H.sapiens      ARVCHAHPTLSEAEAFREANLAASFGKSINF-    511
C.elegans      ARVCHPHPTLSE--AFREANLAAYCGKAINNV    495
               ***** ******  *********  **:**
```

Supplemental Figure 1. Sequence homology of human and *C. elegans* DLD proteins. Different interfaces and domains are specified. The mitochondrial targeting sequence (MTS) would be truncated following transport into the mitochondrial matrix and is absent in the active ketoacid dehydrogenase complexes.

Supplemental Figure 2

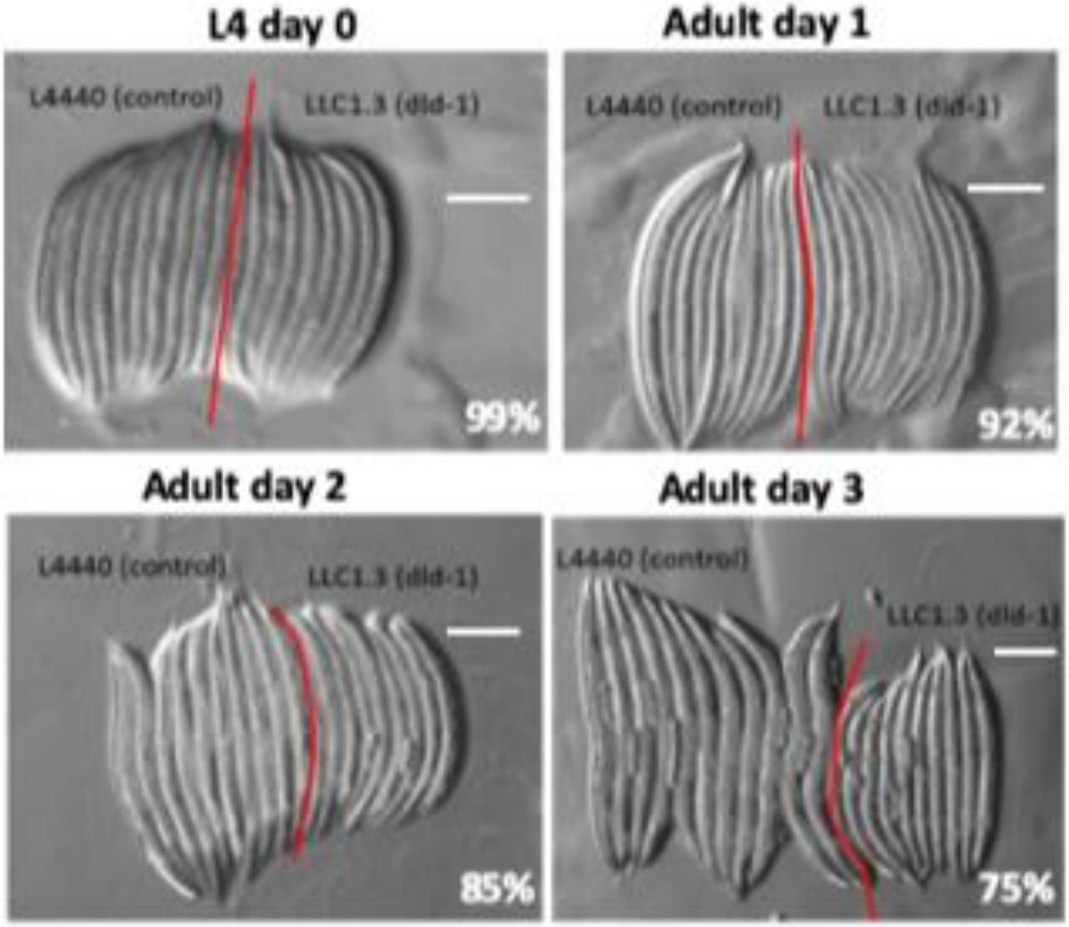

Supplemental Figure 2. DLD-1 knockdown by feeding RNAi in *C. elegans* from egg hatching reduces adult growth. At L4 stage, worms were not significantly different in size. Their reduced size differential becomes more evident as adults and reaches statistical significance relative to wild-type (N2) worms at adult day 3 for the full dose *dld-1(RNAi)* worms. Note that adult worm growth is not as evident when comparing images because of the indicated sequential reductions in size of the images. The white scale bar is a constant 30 pixels in all panels.

Supplemental Figure 3

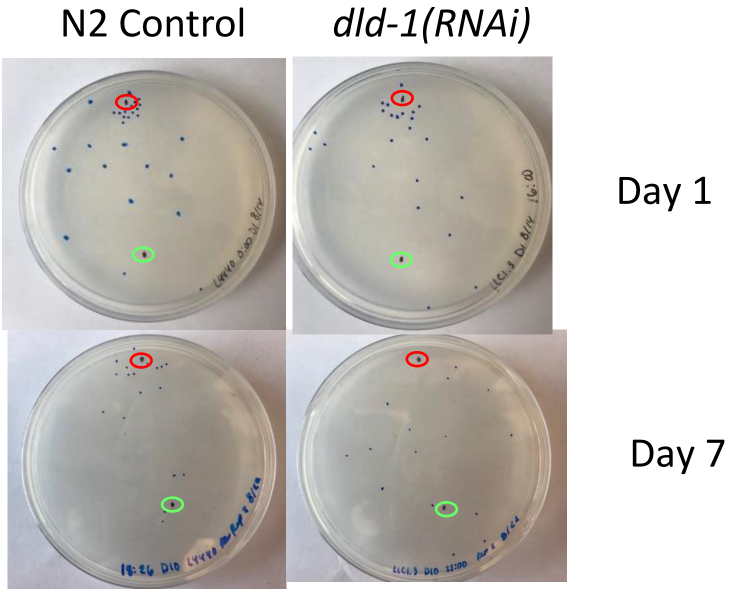

Supplemental Figure 3. Chemotaxis assay performance details. Chemoattraction was assayed by placing worms on nematode growth media agar plates at the origin, highlighted with a green circle, and isoamyl alcohol, a diffusible chemoattractant, 5 cm away in a well, identified by the red circle. Worms, identified as blue-black spots, were allowed to freely migrate for 1 h, after which time each worm's distance from the chemoattractant well was measured on different days of adulthood. At day 7, while N2 worms still migrated with some efficacy towards the chemoattractant the *dld-1(RNAi)* knockdown worms moved in largely random directions.

Supplemental Figure 4

A **B**

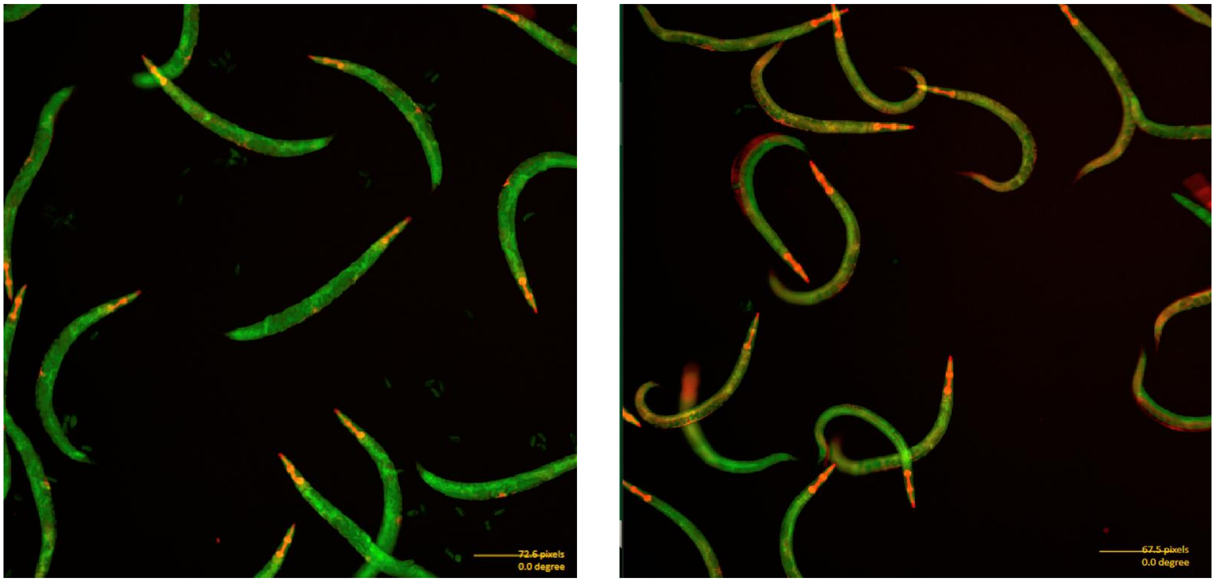

Supplemental Figure 4. Determination of mitochondrial membrane potential with relative TMRE uptake. (A) Wild-type (N2) worms expressing a COX4::GFP after exposure to 1 µM TMRE and subsequent washout **(B)** Full-dose *dld-1(RNAi)* worms expressing COX4::GFP following exposure to TMRE and subsequent washout.

Supplemental Figure 5

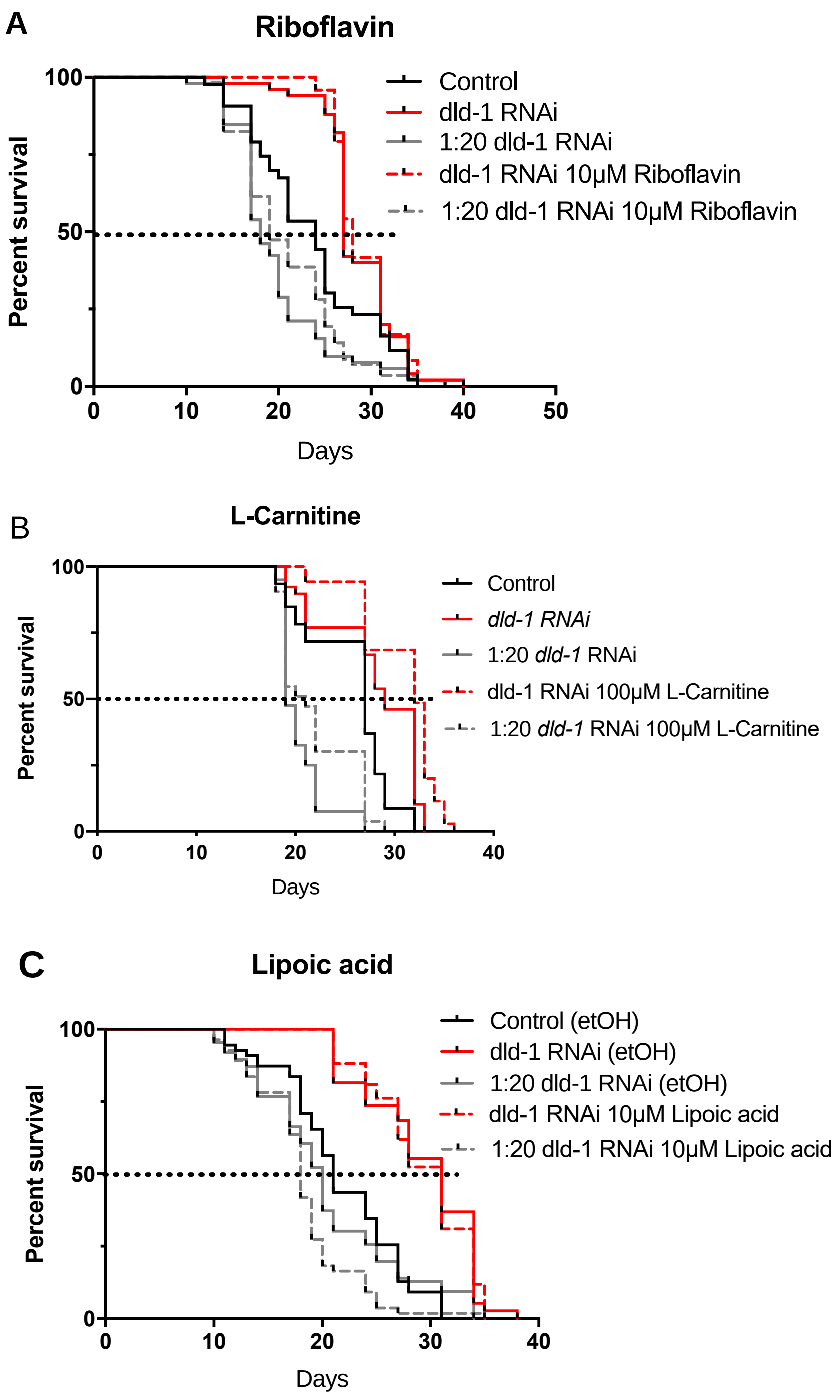

Supplemental Figure 5. Survival analyses of *dld-1(RNAi)* worms treated with riboflavin, L-carnitine, and lipoic acid. (A) Riboflavin at 10 μM, (B) L-carnitine at 100 μM), and (C) Lipoic acid at 10 μM did not significantly rescue the shortened lifespan of 1:20 *dld-1(RNAi)* worms or the extended lifespan of full-dose *dld-1(RNAi)* worms. Drug-treated survival experiment details are otherwise the same as described in Figure 6.

Supplemental Figure 6

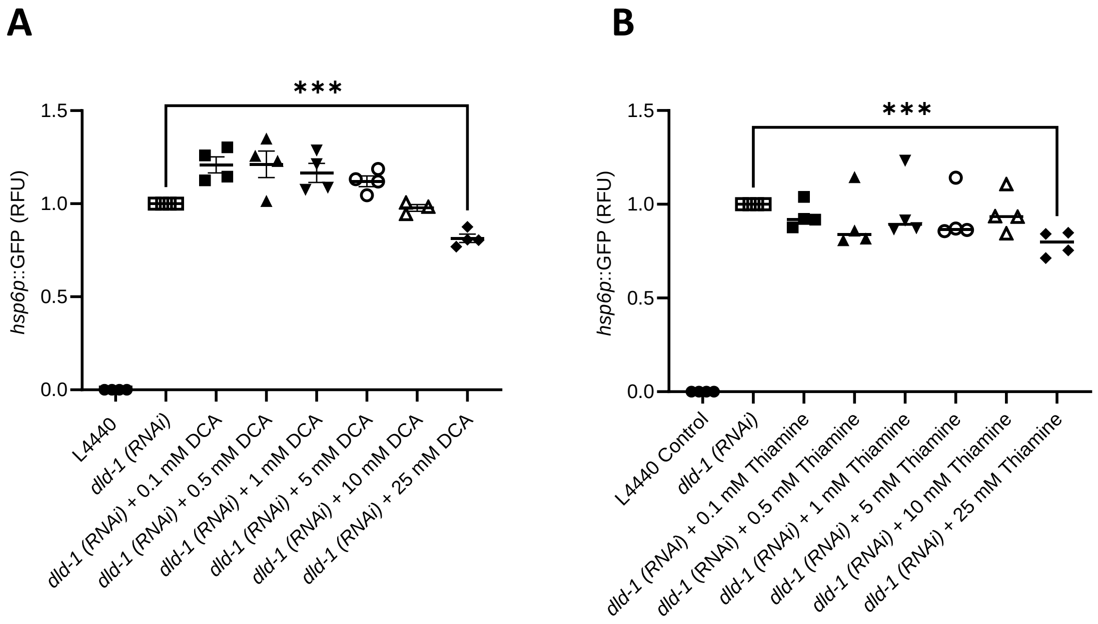

Supplemental Figure 6. UPR^{mt} dose response curves for DCA and thiamine. Full dose *dld-1(RNAi)* worms were treated with 0.1 to 25 mM concentrations of either of the two drugs from hatching, that demonstrated efficacy at reducing the UPR ^{mt}, a prominent phenotype of the worms, in four biological replicates. Each data point represents the mean of ~300 worms normalized to the value for the untreated worms ± the SEM. For both drugs, the trend became statistically significant with the 25 mM dose (****P* < 0.001).

Supplemental Table 1

**Water Soluble Drugs**	**Size**	***hsp6p*::GFP**
Nicotinic Acid (1 mM)	No Effect	No Effect
Nicotinamide (200 µM)	No Effect	No Effect
Riboflavin (10 µM)	Increase	Increase
Thiamine (25 mM)	Increase	Decrease
L-Carnitine (100 µM)	No Effect	No Effect
Folinic Acid (10 µM)	No Effect	Increase
Glucose (10 mM)	No Effect	Increase
DCA (25 mM)	No Effect	Decrease
Cysteamine (100 µm)	No Effect	No Effect
Bitartrate (100 µM)	No effect	Decrease
NAC (2.5 mM)	No Effect	No Effect
AICAR (500 µM)	No Effect	Decrease
Hydralazine (200 µM)	Increase	No Effect
Lithium Chloride (10 mM)	No Effect	Decrease
Cycloheximide (2 µM)	No Effect	Increase
Arginine (10 mM)	No Effect	Increase
Taurine (800 µM)	Increase	Decrease
Taurine (8 mM)	No Effect	No Effect
**DMSO Soluble Drugs**	**Size**	***hsp6p*::GFP**
Epicatechin (10 nM)	No Effect	No Effect
Resveratrol (50 µM)	Increase	Increase
**Ethanol Soluble Drugs**	**Size**	***hsp6p*::GFP**
Rapamycin (2.5 nM)	No Effect	No Effect
Probucol (5 µM)	No Effect	No Effect
Lipoic Acid (10 µM)	No Effect	Increase
Vitamin E (250 µM)	No Effect	Increase

Supplemental Table 1. Drugs tested at the noted concentration for their effects on the growth of *dld-1(RNAi) C. elegans* for their effects on growth at adult day 3 and on the expression of GFP under the control of the HSP6 promoter at adult day 2.

Full Unedited Complete Gel for Figure 2A. Four separate images of the same gel. (A) The gel was probed with anti-DLD (red fluorescence) with fluorescent green molecular weight markers in lane 4. Lanes 1-3 were loaded with the digest of worms fed: (lane 1) full *dld-1(RNAi)*, (lane 2) 1:20 *dld-1(RNAi),* (lane 3) control plasmid L4440. **(B)** Because the electrophoretic mobility of the tubulin and DLD-1 are nearly identical, the gel in panel A was stripped and probed with anti-tubulin (red fluorescence) to serve as a *C. elegans* protein loading standard. **(C)** The image in (A) was flipped and the gel converted to gray scale for integration using Image J **(D)** The image in (B) was flipped and the gel converted to gray scale for integration using Image J.

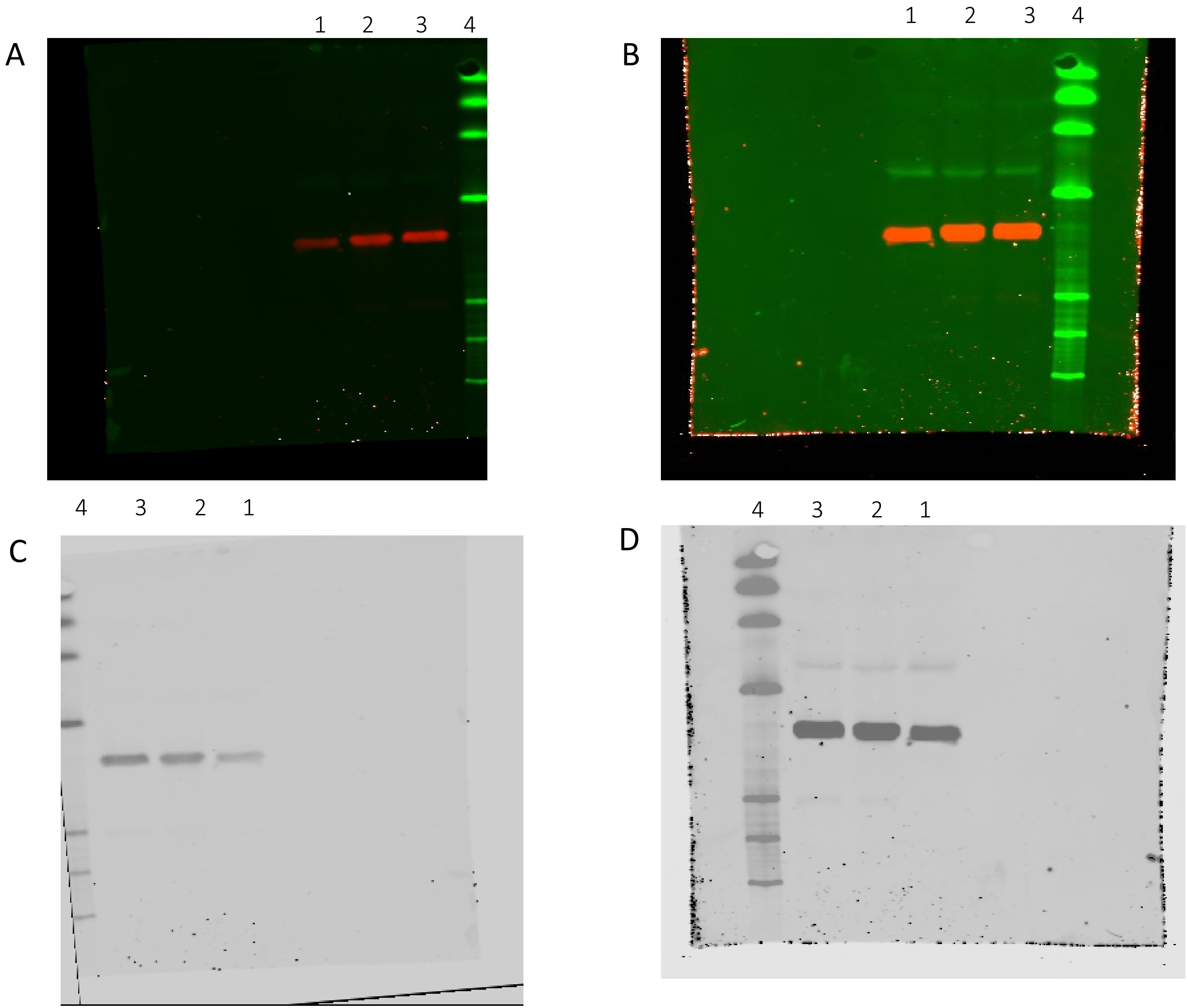

Supplement: Supplemental data [file jciinsight-7-156222-s214.pdf]
